# EU mitigation potential of harvested wood products

**DOI:** 10.1186/s13021-015-0016-7

**Published:** 2015-02-25

**Authors:** Roberto Pilli, Giulia Fiorese, Giacomo Grassi

**Affiliations:** European Commission, Joint Research Centre, Institute for Environment and Sustainability, Via E. Fermi 2749, I-21027 Ispra, VA Italy

**Keywords:** Harvested wood products (HWP), Carbon, FAOSTAT, LULUCF

## Abstract

**Background:**

The new rules for the Land Use, Land Use Change and Forestry sector under the Kyoto Protocol recognized the importance of Harvested Wood Products (HWP) in climate change mitigation. We used the Tier 2 method proposed in the 2013 IPCC KP Supplement to estimate emissions and removals from HWP from 1990 to 2030 in EU-28 countries with three future harvest scenarios (constant historical average, and +/−20% in 2030).

**Results:**

For the historical period (2000–2012) our results are consistent with other studies, indicating a HWP sink equal on average to −44.0 Mt CO_2_ yr^−1^ (about 10% of the sink by forest pools). Assuming a constant historical harvest scenario and future distribution of the total harvest among each commodity, the HWP sink decreases to −22.9 Mt CO_2_ yr^−1^ in 2030. The increasing and decreasing harvest scenarios produced a HWP sink of −43.2 and −9.0 Mt CO_2_ yr^−1^ in 2030, respectively. Other factors may play an important role on HWP sink, including: (i) the relative share of different wood products, and (ii) the combined effect of production, import and export on the domestic production of each commodity.

**Conclusions:**

Maintaining a constant historical harvest, the HWP sink will slowly tend to saturate, i.e. to approach zero in the long term. The current HWP sink will be maintained only by further increasing the current harvest; however, this will tend to reduce the current sink in forest biomass, at least in the short term. Overall, our results suggest that: (i) there is limited potential for additional HWP sink in the EU; (ii) the HWP mitigation potential should be analyzed in conjunction with other mitigation components (e.g. sink in forest biomass, energy and material substitution by wood).

## Background

Forests and the forest sector play a relevant role in the carbon (C) cycle and can significantly contribute to the mitigation of global climate change [1,2]. Specifically, forest-related mitigation options include a change in C stocks - which reflects emissions or removals of CO_2_ from the atmosphere - and a substitution effect. Changes in C stocks can happen both within the forest pools (living biomass, dead wood, litter and soil) and in the harvested wood products (HWP) pool [3]. Substitution effects can occur when wood products replace materials or energy (e.g., concrete or fossil fuels) [4,5]. Given the trade-offs between the forest sink at large scale, wood products and bioenergy (see for example [6-9]), the most effective forest mitigation strategy is the one that maximizes the sum of various mitigation components.

### Accounting approaches for HWP

The role of HWP in mitigating GHG emissions has been recognized only recently by the Kyoto Protocol (KP). For the first KP commitment period (2008–2012) it was assumed that the annual amount of C leaving the HWP pool equals the annual C inflow to the pool. This means that all C in the harvested biomass is oxidized at the time of harvest. In reality, wood-based materials may emit C over a long time frame. Depending on the balance between C inflow and outflow, and the corresponding C stock change, the HWP pool may indeed act as a sink or as a source of CO_2_. For this reason, for the second KP commitment period (2013–2020) accounting rules have been changed to include explicitly C stock changes in the HWP pool [10].

Carbon stock changes in HWP depend on several factors such as the amount of harvest, the final products and their end use, the service life of products, and the disposal/recycling or use as fuel at the end of service life [11]. Different approaches exist to account for C stock changes in the HWP pool [3,12,13]. In particular, the IPCC production accounting approach [3] has been applied to many individual countries or regions such as Northern US [14] and Ireland [13]. More recently, the 2013 Revised Supplementary Methods and Good Practice Guidance Arising from the Kyoto Protocol (2013 IPCC KP Supplement in the following) defined the methods, named Tiers 1 to 3, to be used under the KP according to the level of detail and of accuracy of the available data [15]. The principles behind this new method are the same as applied in Rüter [16] to estimate the current and future HWP emissions/removals in EU countries.

### Future mitigation potential of forests

At the EU level (i.e. 28 Member States) forests cover about 165 Mha [17]. The EU forest area increased by 5% compared to 1990 and now equals about 37% of total EU area. Most of the EU forest area (83%) is available for wood supply [18] and EU is one of the main world producer of roundwood, with about 405 million m^3^ in 2010. Nevertheless, on average only 64% of the EU forest annual increment is removed from the growing stock by fellings^a^. As a consequence, forests in the EU are a major carbon sink: between 1990 and 2012 the average annual forest sink was about 435 million tons of CO_2_ [17]. This corresponds to about 9% of 2010 EU emissions in the same period. While this forest sink has been approximately stable in the past two decades, possible first signs of saturation have been suggested [19], also based on a reported decline in stem volume increment possibly related to forest aging.

In addition, several analyses suggest a significant increase in harvest removals at EU level for the next few decades, mainly due to increasing wood demand for renewable energy production. The EU Reference Scenario 2013 [20] expects an increase of harvest by 17% in 2030 compared to 2005, associated to a decline by about 30% of the forest sink. In the same Reference Scenario, based on the 2010 statistics from EUROSTAT and including the effect of the on-going economic downturn, it is expected that in 2030 forest wood used for energy will increase by 41% while forest used for wood products will increase only by 13%, with respect to 2005 [20]. Mantau et al. [21], for the EU (without Croatia), estimated an increase of the total demand for wood biomass (including biomass power plantations, pellets, etc.) from almost 800 million m^3^ in 2010 to nearly 1400 million m^3^ and 1200 million m^3^ under the A1 and B2 IPCC 2000 scenario, respectively. Considering the B2 IPCC 2000 scenario (i.e., assuming a modest GDP growth rates in Europe), EFI-GTM models projects from 2010 to 2030 a 15% increase of total consumption for wood products in UNECE countries in Europe, including Russia [5]. This trend appears mainly driven by the consumption of wood fuel, which is expected to increase by 35% from 2010 to 2030. Similar trends are suggested also by other studies, e.g. the EU blueprint for forest-based industries [22] and Böttcher et al. [7]. Overall, the expected increase in harvest rate at EU level will heavily influence both the forest sink and the carbon stock changes in the HWP pool.

### Objectives of this study

In order to optimize the overall forest mitigation potential, tools are needed to estimate the specific mitigation potentials of forest management (e.g., [23]), energy uses (e.g., [24]) and wood products. The aim of this paper is to describe a tool for estimating the present and future C stock changes in the HWP pool at EU level, as part of a comprehensive modelling framework for the forest sector [25]. Specifically, following the methods in the 2013 IPCC KP Supplement [15], we estimated HWP emissions/removals for EU countries (with the exception of Malta and Cyprus) (i) for the historical period (from 1990 to 2012) and (ii) until 2030. In this second case we assessed the impact of different harvest amounts on the HWP mitigation potential. Three different scenarios for future total harvest (constant historical average, and +/−20% in 2030) were analyzed. Furthermore, for the constant harvest scenario, the impact of three different distributions of future harvest between each commodity was explored.

### Accounting method and activity data in this study

According to the 2013 IPCC KP Supplement [15], for the second commitment period of the KP, countries have to account the C stock change on the HWP pool from domestic harvest (i.e., the trees harvested in the reporting countries) following one of these methods: (i) instantaneous oxidation (Tier 1); (ii) a default method proposed in the same supplement (Tier 2); (iii) country-specific methods (Tier 3).

The first approach ignores the changes of the HWP stock, with the consequent assumption that all wood is instantaneously burned. This method has been used in the first commitment period of KP. The Tier 2 method applies first order decay functions based on default half-lives numbers distinguished between the main semi-finished wood products (i.e. sawn wood, wood panels and paper) and defined by the international classification system of forestry products. All the countries which proposed a “reference level” for forest management in the second commitment period of the KP (i.e., all the EU countries) have to use at least this approach. If more detailed data and methodologies are available, a country-specific method can be used (Tier 3 approach).

In order to be consistent across all EU countries, the Tier 2 method is used in the following analysis. From the FAOSTAT database^b^ [26] we collected consistent, transparent and verifiable activity data on HWP (production, import and export) for each country. The activity data required for the Tier 2 method are:

- Roundwood removals^c^
- Industrial Roundwood (IRW) i.e., the portion of roundwood removals used for the production of wood commodities
- Sawn wood (SW), wood panels (WP) and paper and paperboard (PP), i.e. the three semi-finished wood products categories.

These categories can also be distinguished between coniferous and not-coniferous (i.e., broadleaves). The historical FAOSTAT data are complete from 1961 for 17 out of 28 countries and largely missing for 2 countries (Malta and Cyprus). For some countries, such as Luxembourg or Eastern EU countries, only data for the last 10 – 20 years are available (Table 1).

**Table 1:** **Activity data analysis**: **for 28 EU countries**

**Countries**	**A. FAOSTAT**	**B. Specific data sources in comparison with FAOSTAT**				**C. Possible explanations for differences between A and B**	**D. Corr. factors**
	**Available since**	**FRA CR**	**NIR**	**NFI**	**FMRL**		
**Austria**	1961	=	↑	X	↑	**Bark fraction**	1.15
**Belgium**	2000	↓	↓		↓	**Accounting methods**	-
**Bulgaria**	1961	=			=		-
**Croatia**	1992	X			↑	**Forest residues & bark**	1.10
**Cyprus**	N. A.	X					-
**Czech Rep**	1993	=	X		↑	**Forest residues & bark**	1.10
**Denmark**	1961	=			X	**Forest residues**	-
**Estonia**	1992	X	=^1^		=^1^	**Forest residues,** **bark & other**	1.10
**Finland**	1961	↑	↑		↑	**Forest residues,** **bark & other**	1.10
**France**	1961	=	↓		=		-
**Germany**	1961	↑	=		↑	**Forest residues,** **bark & other**	1.44^2^
**Greece**	2007	↑			↑	**Bark fraction**	1.15
**Hungary**	1961	X	↑		↑	**Bark fraction & forest residues**	1.20^3^
**Ireland**	1961	X			↑	**Bark fraction**	1.10
**Italy**	1961	=		X	↑	**Forest residues,** **bark & other**	1.57^4^
**Latvia**	1992	X	↓		↓	**Bark fraction**	1.12
**Lithuania**	1992	X			↑	**Bark fraction**	1.12
**Luxemb**.	2000	=			↑		-
**Malta**	N. A.	X					
**Netherlands**	1961	X			↑	**Bark fraction**	1.15-1.18
**Poland**	1961	↑			=	**Bark fraction**	1.20
**Portugal**	1961	↑			=	**Bark fraction**	1.25-1.18
**Romania**	1961	=	↑		↑	**General CF**	1.23^5^
**Slovakia**	1993	X	=		=	**Bark fraction**	1.10-1.12
**Slovenia**	1993	X			↑	**Bark fraction**	1.17-1.13
**Spain**	1961	↑			↑		1.10
**Sweden**	1961	↑			↑	**Bark fraction & forest residues**	1.14
**UK**	1961	X			↑	**Bark fraction**	1.14-1.12

The table reports: A. The first year from which FAOSTAT data are available; B. the additional data sources considered by this study, including: the 2010 Forest Resource Assessment country’s report (FRA CR), the 2013 National Inventory Reports (NIR), the last National Forest Inventory (NFI, when public available) and the Submission for Forest Management Reference Level (FMRL).

Symbols highlight if the amount of harvest reported by these specific data sources are, on average: equal (=), higher (↑), lower (↓) or not comparable (X, because of different time scales or other reasons) as compared to the FAOSTAT data. C. Possible differences between FAOSTAT and the other specific data sources. D. The correction factors applied to the original FAOSTAT data, mostly based on a correction for bark. The bark’s correction factor (based on data from the literature, when available at country level) was applied when, comparing FAOSTAT data with other sources (mainly the 2010 FRA Country Report), we argued that the volume reported by original FAOSTAT data were under-bark.

^1^the NIR 2013 reports the same values reported by FAOSTAT since 2003.

^2^average general correction factor (accounting for bark and other corrections) applied to original FAOSTAT data from 2000 to 2012; the CF varied year by year, assuming that the figures reported by the Submission for FMRL represent the correct estimates (Joachim Rock, pers. com).

^3^bark’s CF applied only to the industrial roundwood compartment.

^4^average general correction factor (accounting for bark, forest residues and other corrections, suggested by [28] and by [23].

^5^average general correction factor suggested by NIR 2013 [28].

We compared FAOSTAT data with other available data sources, including: (i) the 2010 Forest Resource Assessment country’s reports [27]; (ii) the 2013 National Inventory Reports [28] submitted by each country to the United Nations Framework Convention on Climate Change (UNFCCC); (iii) the last National Forest Inventories (NFIs, when public data were available) and (iv) the countries’ submissions for Forest Management Reference Level [29]. These last documents, submitted to UNFCCC in 2011, generally provided additional information on the historical (i.e., until 2008) and future harvest rate, used to assess the FMRL for the second commitment period of the KP. All data were preliminary harmonized, taking into account over and under bark correction factors and other possible corrections due to the accounting of forest residues or under- overestimates reported by official statistics. A comparison of the different data sources and a summary of the corrections applied to original FAOSTAT data are reported in Table 1.

**Figure 1:**
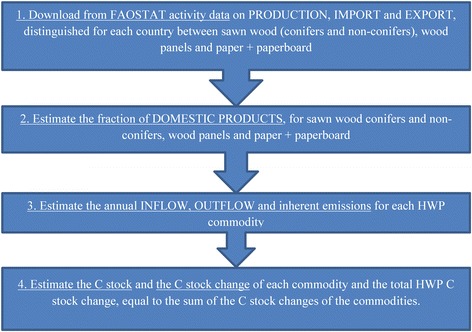
**IPCC Tier 2 steps:** **main steps applied to the HWP pool to estimate the total C stock and C stock change according to the Tier 2 method** **[** 15 **].**

The 2013 IPCC KP Supplement Tier 2 method is basically a flux data method where estimates of net emissions are derived from a stock change calculation applied to products derived from domestic harvest, i.e., imported HWP are excluded. To implement this method, it is first necessary to estimate the annual fraction of the industrial roundwood (sawn wood and wood based panels) and wood pulp commodities coming from domestic harvest. According to IPCC, the C stock included in fuelwood is immediately released to the atmosphere. The main steps on this method are summarized in Figure 1.

We assume that all domestic harvest derives from forest management, thus we do not differentiate harvest coming from afforestation activities. Indeed, due to the low rate of afforestation and the young age of the afforested lands, the share of harvest potentially provided by this category is generally negligible [30], with few exceptions [31]. According to 2013 IPCC KP Supplement [15], the instantaneous oxidation method must be applied to harvest from deforestation. Due to the lack of reliable data, in this study we did not consider any harvest from deforestation and thus did not assess the related C emissions. Moreover, due to the relative small amount of area affected by deforestation in EU, equal on average to 109 kha yr^−1^ [32] for the period 2008 – 2012 over a total FM area equal to 140,030 kha (as considered in this study), we can assume that the total amount of harvest provided by deforestation is negligible compared with that of the forest management area. Finally, because this work is part of a more comprehensive modelling framework for the forest sector [25], we plan to include deforestation in future developments.

The share of domestically produced SW, WP and PP for the domestic production (DP) is computed (step 1 of Figure 1) considering the production, import and export of the feedstock commodities, IRW and Pulp. When the amount of DP in each commodity has been estimated (step 2 of Figure 1), it is then possible to calculate the associated flows of carbon (step 3 of Figure 1).

The C stock of each HWP category, in each year, is estimated by applying a first order decay function taking into account the C outflow and inflow from and to each category, as reported in the Methods (see the subsection on First Order decay functions).

To complete the assessment, we account also for the inherited emissions, i.e. the emissions that occur during the second commitment period from HWP removed from forests prior to the beginning of the second commitment period [15]. We estimate the accumulation of the historic inflow, starting from 1900, assuming that the C inflow until 1961 (or until the first available year, see Table 1) is constant and equal to the average of the first available five years (e.g., generally, 1961–1965), as in [16].

Finally (step 4, Figure 1), the total C stock and C stock change in HWP can be calculated for each country by summing up all the C stocks and C stock changes of all the commodities^d^ (e.g., SW_C_ + SW_NC_ + WP + PP). This approach was applied to estimate for each EU country the historical C stock change in HWP until 2012, i.e., the last year reported by 2013 FAO statistics.

### Future trends

To establish the future mitigation potential of HWP, we first need to estimate how the harvest demand will evolve. To this aim, we used three different harvest scenarios up to 2030:

1. A constant harvest scenario (CH) equal to the average historical harvest (2000–2012);
2. An increasing harvest scenario (CH+) assuming +20% with respect to the CH scenario in 2030 and a linear increase from 2013 to 2030;
3. A decreasing harvest scenario (CH-) assuming −20% with respect to the CH scenario in 2030 and a linear decrease from 2013 to 2030.

**Figure 2:**
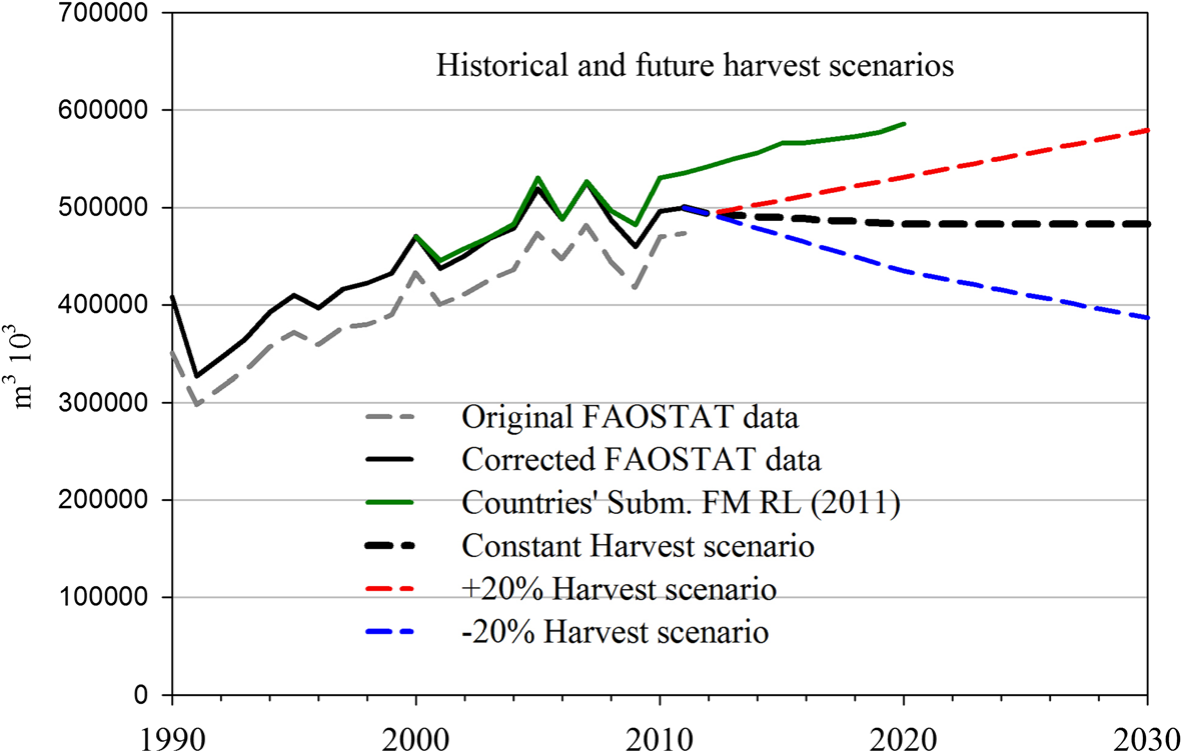
**Total harvest demand: total harvest demand (in m** ^**3**^ **10** ^**3**^ **) for 28 EU countries, based on the historical FAO statistics (until 2012, Original FAOSTAT data) corrected to account for possible under-/over-estimates (Corrected FAOSTAT data used in this paper, see Table** 1 **) and three future harvest scenarios: constant harvest, increasing harvest (+20%) and decreasing harvest (−20%) up to 2030.** A comparison with the harvest provided by countries's Submission FMRL is also reported.

For all these scenarios, the future harvest demand (Figure 2) was defined on the basis of the historical amount of harvest reported by the FAOSTAT data, corrected according to the analysis described above (see Table 1). Of course, we are not considering the fact that harvest projections provided by countries are driven either by projected potential supply of forest biomass and projected demand of forest biomass for the subsequent production of wood products and wood fuel.

The share of domestic feedstock for the production of particular HWP category originating from domestic forests is not directly correlated to the total harvest because of the production processes. For example there may be recycled products, recovered paper, slashes, wood-chips used in wood-based panel production, etc.

To apply the method described above to each harvest scenario, we first calculated the 2008–2012 average C inflow (e.g., the C inflow of the last five years) and then we applied a constant, increasing (+20%) and decreasing (−20%) variation rate to this average, according to each harvest scenario. This implicitly assumes to use the historical distribution of the total harvest between each commodity and to vary this distribution proportionally to the future harvest scenario. Because this is the same approach used in Rüter [16] and applied by the majority of EU countries for the submissions for FMRL, we considered this method as Approach 0 (AP0).

For the constant harvest scenario, we also explored two other possible approaches (AP1 and AP2). The aim of these two approaches is to explore, through statistical correlations among the variables requested by the default Tier 2 method, the impact of deviating from the AP0 assumption that the future distribution of the total harvest among each commodity is constant and equal to the historical distribution. All these approaches follow the IPCC 2013 Tier 2 method.

Approach 1 (AP1) starts at step 1 described in the Methods (Figure 1). With AP1 we estimate the future distribution of the total harvest between each commodity (i.e., SW, WP and PP) and the amount of IRW and pulp production, import and export (this is only aimed to calculate the annual fraction of domestic production).

Approach 2 (AP2) starts at step 2 described in the Methods (i.e., the estimate of the domestic production of the 4 commodities).

To this aim, for each country, we first looked for possible statistical correlations between the available information on the total harvest or the time (e.g., years) and the SW, WP, and PP production (for AP1) or domestic production (for AP2). The rationale is that the correlations that best describe the past data could then be used to estimate the future evolution of each variable, applying the default Tier 2 Method.

## Results and discussions

### Historical and future harvest rate

The total amount of harvest at EU level, reported by FAOSTAT and corrected to account for bark’s fractions or other possible over- or under-estimations, is shown in Figure 2.

Based on FAOSTAT data, more than 60% of the total wood harvest (measured as the 2000–2012 average) is provided by only five countries: Sweden (17% of the total), Germany (14%), Finland (12%), France (12%) and Poland (8%). Eight countries (Austria, Czech Republic, Romania, Spain, Latvia, Portugal, Italy, UK) contribute to another 25%, each with 2-4% share of the total EU harvest. The remaining 12% of wood is harvested in 13 countries, each contributing on average less than 1%. From these figures, it emerges that the estimates and data regarding the first five countries are extremely relevant with respect to any analysis of the forest sector in general and of the HWP sector in particular. For example, the input data for Germany extracted from the FAOSTAT database (based on production statistics) is different from other data sources, such as the NIR (derived from forest inventory).

Between 2000 and 2011 (for some countries data prior to 2000 are missing, see Table 1), FAOSTAT original data show the same trend reported by the country’s submissions for FMRL [29]. However, the total amount of harvest reported by the submissions is on average 10% higher than FAOSTAT. In most cases, this difference is due to the bark fraction, which sometimes is not accounted for in FAOSTAT. Furthermore, other specific issues that vary country-by-country can explain the difference (Table 1). When FAOSTAT data are corrected according to additional information from FRA 2010 Country Reports and to other available data (i.e., from the last NIRs), then the difference with data from FMRL decreases to less than 2%. If we look specifically at 2010 and 2011, the difference between the two data sources is higher (about 7%). This difference is related to the fact that in FMRL submissions the years 2010 and 2011 were already part of the “projected” estimates, i.e. based on future assumptions and not on statistics.

The 2010 harvest rate based on FAOSTAT corrected data is about 72% of the potential forest woody biomass resource (686 million m^3^) estimated by Mantau et al. [21] according to the IPCC 2000 scenario A1, assuming a medium mobilization scenario. Reducing this amount by forest residues (118 million m^3^, not accounted by FAOSTAT), the resulting amount (568 million m^3^) is about 14% higher than our FAOSTAT corrected data. This difference appears related to the fact that Mantau et al. [21] did not consider the effect of the last economic crisis.

The 2030 harvest rate applied in our study is equal to 482 million m^3^, 386 million m^3^ and 579 million m^3^, assuming respectively a constant, decreasing and increasing harvest rate. This last value is similar (about 6% lower) to the estimates used by the G4M and GLOBIOM models in the EU reference scenario [20].

### Approach 1 (AP1)

A synthesis of the results based on AP1 is presented in Table 2, for each country and commodity. Applying the AP1 to the historical FAOSTAT data– corrected according to our analysis - shows that, in most countries, the production of *SW*, *WP* and *PP* is statistically correlated with the total roundwood production and with time. This means that, for some countries, (i) there is a statistical correlation between time (i.e., years) and/or total roundwood production and the SW, WP and PP production and (ii) in most cases, we highlighted a temporal trend (increasing or decreasing) on the data series (i.e., a correlation with time) above all for the PP and WP production.

**Table 2:** **Results from AP1**: **For each key variable the table reports** **the independent variable** (**i.e**., **total roundwood** (**RW**) **or time** (**t**)) **applied to Eq**. 6 **followed by the coefficient of determination** ***R*** ^***2***^ **of the linear regression model**

	**To calculate the C stock and C stock change**				**To calculate the share of domestically produced IRW and Pulp**								
**Country**	**PRODUCTION**				**PRODUCTION**			**IMPORTS**			**EXPORTS**		
	**SWc**	**SWnc**	**WP**	**PP**	**IRWc**	**IRWnc**	**Pulp**	**IRWc**	**IRWnc**	**Pulp**	**IRWc**	**IRWnc**	**Pulp**
**Austria**	f(RW) 0.79	Avg	f(RW) 0.82	f(RW) 0.76	f(RW) 0.95	IRW-IRW_C_	f(t) 0.85	f(t) 0.87	f(t) 0.72	f(t) 0.92	f(t) 0.79	Avg	f(t) 0.95
**Belgium**	Avg	Avg	f(RW) 0.78	f(RW) 0.75	f(RW) 0.94	IRW-IRW_C_	Avg	Avg	Avg	Avg	f(t) 0.64	Avg	Avg
**Bulgaria**	f(RW) 0.78	Avg	Avg	f(RW) 0.71	f(RW) 0.94	IRW-IRW_C_	f(RW) 0.69	Avg	f(t) 0.65	f(t) 0.95	f(t) 0.68	f(t) 0.67	f(t) 0.76
**Croatia**	Avg	Avg	Avg	f(RW) 0.89	IRW_NC_-IRW	f(RW) 0.97	Avg	Avg	Avg	Avg	Avg	Avg	Avg
**Cyprus**													
**Czech Rep**	f(RW) 0.86	Avg	f(RW) 0.86	f(RW) 0.81	f(RW) 0.99	IRW-IRW_C_	f(RW) 0.95	Avg	Avg	Avg	Avg	Avg	Avg
**Denmark**	Avg	Avg	Avg	f(t) 0.91	IRW_NC_-IRW	f(t) -0.82	Avg	f(t) 0.70	Avg	f(t) 0.97	Avg	Avg	f(t) 0.92
**Estonia**	Avg	f(t) 0.72	Avg	f(t) 0.73	f(RW) 0.99	IRW-IRW_C_	f(t) 0.88	Avg	Avg	f(t) 0.93	Avg	Avg	f(t) 0.90
**Finland**	f(RW) 0.76	Avg	Avg	f(t) 0.96	f(RW) 0.93	IRW-IRW_C_	f(t) 0.69	Avg.	f(t) 0.72	f(t) 0.86	Avg	Avg	f(t) 0.95
**France**	f(RW) 0.85	Avg	f(t) 0.85	f(RW) 0.80	f(RW) 0.83	IRW-IRW_C_	f(t) 0.95	Avg	Avg	f(t) 0.95	Avg	Avg	f(t) 0.91
**Germany**	f(RW) 0.77	f(t) 0.75	f(t) 0.91	f(t) 0.97	f(RW) 0.99	IRW-IRW_C_	f(t) 0.84	Avg.	Avg	f(t) 0.95	Avg	f(t) 0.66	f(t) 0.91
**Greece**	Avg	Avg	f(t) 0.82	f(t) 0.91	Avg	IRW-IRW_C_	Avg	f(t) 0.66	Avg	f(t) 0.88	Avg	Avg	f(t) 0.75
**Hungary**	Avg	Avg	f(t) 0.85	Avg	Diff.	Avg	Avg	Avg	Avg	f(t) 0.9	Avg	Avg	f(t) 0.93
**Ireland**	f(RW) 0.96	f(t) 0.74	f(RW) 0.81	Avg	f(RW) 0.99	IRW-IRW_C_	Avg	Avg	Avg.	f(t) 0.92	f(t) 0.72	Avg	Avg
**Italy**	Avg	f(t) 0.69	f(t) 0.88	f(t) 0.94	IRW_NC_-IRW	f(t) -0.81	Avg	Avg	Avg.	f(t) 0.93	Avg.	Avg.	f(t) 0.92
**Latvia**	f(RW) 0.92	Avg	Avg	f(t) 0.91	f(RW) 0.94	IRW-IRW_C_	Avg	Avg	Avg	f(t) 0.94	Avg.	Avg.	f(t) 0.77
**Lithuania**	Avg	f(RW) 0.70	f(t) 0.88	f(t) 0.94	f(RW) 0.80	IRW-IRW_C_	Avg	Avg	f(t) 0.70	f(t) 0.95	Avg	f(t) 0.66	f(t) 0.95
**Luxemb**.	Avg	Avg	f(t) 0.72	f(t) 0.66	IRW_NC_-IRW	f(RW) 0.96	Avg	Avg	Avg	Avg	Avg	Avg	Avg
**Malta**													
**Netherl**.	f(t) 0.70	Avg	f(t) 0.84	f(t) 0.92	f(RW) 0.83	IRW-IRW_C_	f(t) 0.81	Avg	Avg	f(t) 0.98	Avg	Avg	f(t) 0.91
**Poland**	f(t) 0.74	f(t) 0.66	f(RW) 0.95	f(RW) 0.96	f(RW) 0.97	IRW-IRW_C_	Avg	Avg	Avg	Avg	Avg	Avg	f(t) 0.69
**Portugal**	Avg	Avg	f(RW) 0.85	f(RW) 0.69	Avg	f(RW) 0.85	f(t) 0.90	Avg	Avg	f(t) 0.87	Avg	Avg	f(t) 0.84
**Romania**	Avg	f(RW) 0.77	Avg	Avg	f(RW) 0.87	IRW-IRW_C_	Avg	Avg	Avg	f(t) 0.96	Avg	Avg	Avg
**Slovakia**	f(RW) 0.87	f(RW) 0.87	f(RW) 0.78	f(t) 0.66	f(RW) 0.95	IRW-IRW_C_	f(t) 0.83	Avg	Avg	f(t) 0.97	Avg	Avg	f(t) 0.91
**Slovenia**	Avg	Avg	Avg	f(t) 0.74	f(RW) 0.94	IRW-IRW_C_	Avg	Avg	Avg	f(t) 0.9	Avg	Avg	f(t) 0.88
**Spain**	f(t) 0.78	Avg	f(t) 0.91	f(t) 0.98	IRW_NC_-IRW	f(t) 0.95	f(t) 0.97	Avg	Avg	f(t) 0.84	Avg	Avg	f(t) 0.79
**Sweden**	f(RW) 0.86	Avg	Avg	Avg	f(RW) 0.99	IRW-IRW_C_	Avg	Avg	Avg	f(t) 0.89	Avg	Avg	f(t) 0.98
**UK**	f(RW) 0.98	f(RW) 0.90	f(RW) 0.97	Avg	f(RW) 0.99	IRW-IRW_C_	Avg	Avg	Avg	f(t) 0.98	Avg	Avg	f(t) 0.73

Where the coefficient of determination r < |0.69|, the average historical values (Avg) was applied. Acronyms stand for: *SW*_*C*_ sawn wood coniferous; *SW*_*NC*_ sawn wood non-coniferous; *WP* wood based panels; *PP* paper and paper boards; *IRW*_*C*_ Industrial Roundwood coniferous; *IRW*_*NC*_ Industrial Roundwood non-coniferous; *IRW* Total Industrial Roundwood.

For 42% (for SW) and 30% (for WP and PP) of the countries, the production of each commodity was estimated using the total RW production as main driver (i.e., as independent variable applied to the linear model described in Materials) and it was therefore directly related to the future harvest demand. In other countries, however, these data were correlated with time (34% and 50% of the countries for WP and PP, respectively) suggesting the existence of an increasing (this was for example the case of the PP production in Spain, and of many other countries) or decreasing (this was the case of the SW_NC_ production in Italy and in few other countries) trend over time. Where no correlation was detected (i.e., 30%, 34% and 20% of the countries for SW, WP and PP production, respectively), we used the constant average of the previous years. In these cases, the production is not statistically correlated to the total amount of harvest but is probably linked to other drivers. This reflects the fact that in some countries the domestic harvest amounts sufficiently supply the demand for producing subsequent products (for these countries we detected a correlation with RW), whereas other countries need to import the feedstock. Of course, this is a quite simplified approach that ignores any technical or economic correlations between forest biomass and production of semi-finished wood products.

### Approach 2 (AP2)

A synthesis of the results based on AP2 is presented in Table 3, for each country and commodity. When using the AP2, the *SW* and *WP* domestic production were estimated using the IRW as independent variable for the linear regression model for 50% and 46% of the countries, respectively. In these cases the DP is estimated from the future amount of wood for non-energy use applied to each scenario. Only in few cases, 10% of the countries for SW and 15% for WP, the domestic production is statistically correlated with time (i.e., a temporal trend on the data series can be clearly highlighted). In the remaining countries, we calculated the average DP of the last years and kept it constant. For the majority of the countries (65%) we also calculated the average PP domestic production of the last years and kept it constant.

**Table 3:** **Results from AP2**: **For each key variable the table reports** (**if Eq**. 6 **was applied**) **the independent variable** (**i.e**., **industrial roundwood** (**IRW**) **or time** (**t**)) **applied and the coefficient of determination** ***R*** ^***2***^ **of the linear regression model**

**COUNTRY**	**SWt**		**SWc**		**SWcn**		**WP**		**PP**	
	**Function**	**R** ^**2**^	**Function**	**R** ^**2**^	**Function**	**R** ^**2**^	**Function**	**R** ^**2**^	**Function**	**R** ^**2**^
**Austria**	f(IRW_t_)	0.82	f(IRW_t_)	0.83	SWt-SWc		f(IRW_t_)	0.69	Average	
**Belgium**			Average		Average		Average		f(IRW_t_)	0.64
**Bulgaria**	f(IRW_t_)	0.68	SW_t_-SW_nc_		f(IRW_t_)	0.82	Average		Average	
**Croatia**			Average		Average		f(IRW_t_)	0.64	f(IRW_t_)	0.86
**Cyprus**	na		na		na		na		na	
**Czech Rep**	f(IRW_t_)	0.78	f(IRW_t_)	0.75	SWt-SWc		f(IRW_t_)	0.82	Average	
**Denmark**			Average		average		Average		Average	
**Estonia**	f(IRW_t_)	0.66	f(IRW_t_)	0.66	SWt-SWc		Average		Average	
**Finland**	f(IRW_t_)		f(IRW_t_)	0.87	SWt-SWc		Average		f(IRW_t_)	0.7
**France**	Average		f(t)	0.72	SWt-SWc		f(t)	0.87	f(t)	0.87
**Germany**	f(IRW_t_)	0.7	f(IRW_t_)	0.75	SWt-SWc		f(IRW_t_)	0.65	f(t)	0.97
**Greece**			Average		Average		f(IRW_t_)	0.92	Average	
**Hungary**			Average		Average		f(IRW_t_)	0.91	Average	
**Ireland**	f(IRW_t_)	0.97	f(IRW_t_)	0.97	SWt-SWc		f(IRW_t_)	0.86	Average	
**Italy**	f(t)	0.76	SWt-SWnc		f(t)	0.81	f(t)	0.86	Average	
**Latvia**	f(IRW_t_)	0.95	f(IRW_t_)	0.95	SWt-SWc		f(t)	0.71	Average	
**Lithuania**	Average		SWt-SWnc		f(IRW_t_)	0.70	f(t)	0.84	Average	
**Luxemb**.			Average		Average		Average		Average	
**Malta**	na		na		Na		Na		na	
**Netherl**.			Average		Average		Average		f(t)	0.74
**Poland**	f(t)	0.75	f(t)	0.75	SWt-SWc		f(IRW_t_)	0.96	Average	
**Portugal**	Average		Average		SWt-SWc		f(IRW_t_)	0.8	f(IRW_t_)	0.8
**Romania**	Average		SWt-SWnc		f(IRW_t_)	0.95	Average		Average	
**Slovakia**	f(IRW_t_)	0.9	f(IRW_t_)	0.92	SWt-SWc		f(IRW_t_)	0.76	Average	
**Slovenia**			Average		Average		Average		Average	
**Spain**	f(IRW_t_)	0.68	f(IRW_t_)	0.61	SWt-SWc		f(IRW_t_)	0.75	f(IRW_t_)	0.88
**Sweden**	f(IRW_t_)	0.86	f(IRW_t_)	0.86	SWt-SWc		Average		f(t)	0.72
**UK**	f(IRW_t_)	0.98	f(IRW_t_)	0.95	SWt-SWc		f(IRW_t_)	0.98	Average	

Acronyms stand for: *SW*_*C*_ sawn wood coniferous; *SW*_*NC*_ sawn wood non-coniferous; *WP* wood based panels; *PP* paper and paper boards.

### HWP mitigation potential

**Figure 3:**
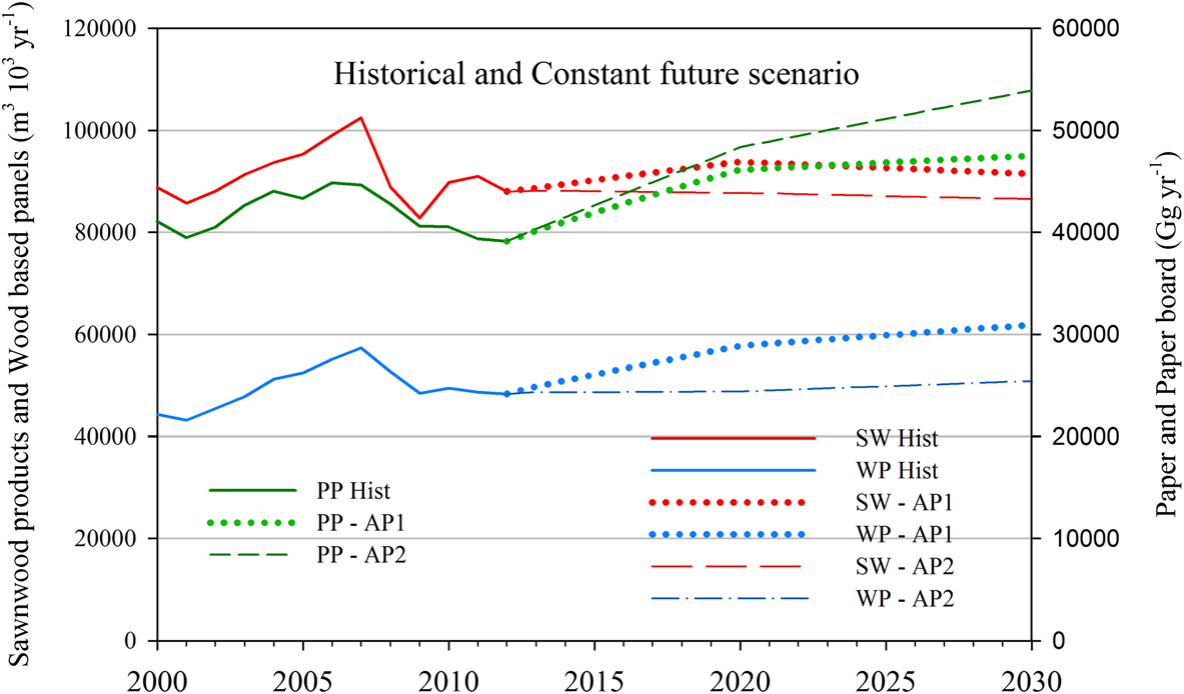
**Total domestic production: total domestic production distinguished between sawn wood products (SW), wood based panels (WP, both in m** ^**3**^ **10** ^**3**^ **yr** ^**−1**^ **, left axis) and paper and paper board (PP, Gg yr** ^**−1**^ **, right axis), estimated applying the IPCC Tier 2 method.** Solid lines show historic data; dotted lines show future trends based on constant harvest scenarios, estimated with the Approach 1 (AP1) and the Approach 2 (AP2).

The historic domestic production of the three HWP commodities, using the IPCC Tier 2 method is shown in Figure 3.

Starting from 2013 and considering the constant harvest scenario, both the AP1 and the AP2 estimate a quite stable SW domestic production. This is due to the fact that: (i) in many countries (42% for AP1 and 50% for AP2) the SW production or DP were estimated using the RW (for AP1) or the IRW (for AP2) as main driver (i.e., a correlation between this commodity and the total harvest was detected); and (ii) where no statistical correlation was detected (i.e., 30% of the countries both for the AP1 and the AP2) a constant production or DP was assumed (of course, this is fully consistent with the constant harvest scenario). In some cases however, including Germany and Poland (i.e., two of the 5 most important EU countries detected by our preliminary analysis on harvest) the SW production or DP is statistically related to time, i.e., a temporal trend was detected, without any correlation with the total amount of harvest. This may also explain the increasing domestic production estimated with AP1 compared with AP2, even with a constant harvest scenario.

The same considerations may explain the increasing WP domestic production estimated with AP1 (+25% compared to the average 2000–2012 values). In this case, for 36% of the countries (including Sweden, Germany and France), the WP production was not statistically correlated with the RW but with time. On the contrary, with AP2 we estimated a constant WP domestic production and only for 4 countries this variable was related to time. For the PP, the 2030 DP is 14% (with AP1) and 18% (with AP2) higher than the 2000–2012 average production. Indeed, with AP1 the PP production was mainly estimated using the time as independent variable and with AP2, we used the time as independent variable for Sweden, Germany and France.

Overall, these results suggest that due to the combined effect of IRW and pulp production, import and export (indirectly affecting the estimates of the DP), variations on the total harvest rate may have different effects on the DP of each commodity and each country. At EU level, the SW and WP DPs generally have a stronger correlation with the total harvest rate but for some important country we detected a temporal trend (i.e., a correlation with time) and no correlation with the total RW or IRW production. For many countries the PP commodity is not related to the total RW production but to other parameters i.e., economic and technical drivers (e.g., recycled paper) not directly considered by our analysis but indirectly included in the variable time. In these cases, the resulting DP is not correlated with the total amount of harvest. Of course, all commodities are not only correlated with the harvest amount but also with the prices development of woody biomass, which also impact the dynamics of import and export.

**Figure 4:**
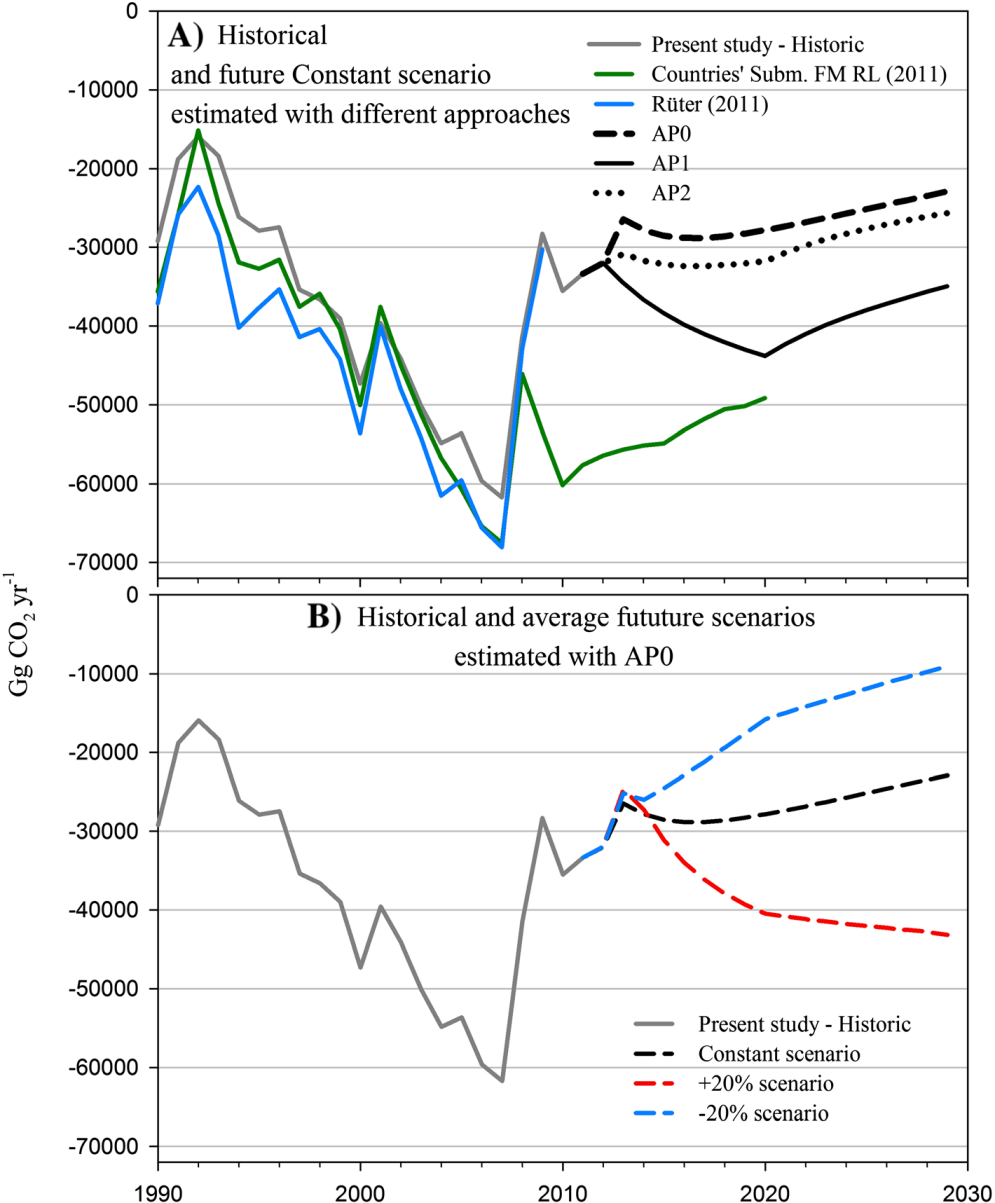
**HWP sink**: **total sink from HWP** **(in Gg CO**_**2**_ **yr** ^**−1**^ **)** **for the historical period** **(until 2012)** **and projections until 2030.** The upper panel **(A)** reports: (i) the estimates provided by our study for the historical period (based on FAOSTAT corrected data); (ii) a comparison with the estimates provided by Rüter [16] and by the country’s Submission for FMRL (2011) and (iii) the future C sink estimated by our study using (a) the AP0, (b) the AP1 and (c) the AP2 approaches. The lower panel **(B)** reports the historical sink estimated by our study until 2012 and the future sink estimated by the AP0 for the constant, increasing (+20%) and decreasing (−20%) harvest scenarios. Please note that for some countries no data was available before 2000.

The historical net sink from HWP estimated by our analysis is reported in Figure 4 (see the upper panel). Until 2009 (i.e., the historical period considered by the submissions for FMRL), we estimated the same trend reported by the countries’ submissions and by Rüter [16]^e^, even if the sink estimated by our study is on average 11% (compared with the submissions for FMRL) and 21% (compared with Rüter’s estimates) lower than these studies (considering the period 1990 – 2008). These differences may be due to (i) different data sources (e.g., [26]) used in Rüter [16] and the present study, respectively, (ii) the application of different carbon conversion factors, (iii) the total harvest rate of some countries and (iv) on the methods used by some countries (i.e., Finland, according to the country’s submission for FMRL, did not consider the import and total production to estimate the fraction of domestic production).

For 2010, our estimates are 70% lower than the estimates reported by the submissions for FMRL. These documents, submitted to UNFCCC in 2011, were based on different assumptions on the future (after 2009) harvest rates. For the same reason, the submissions for FMRL estimated after 2009 a higher sink in 2020, equal to −49,162 Gg CO_2_ yr^−1^, due to the higher harvest rate as compared to our scenarios (see Figure 2).

Overall, for 2030 with a constant harvest scenario, the AP1 estimates higher removals (−34,901 Gg CO_2_ yr^−1^) than the AP2 (−25,652 Gg CO_2_ yr^−1^), mainly due to the higher SW and PP domestic production (see Figure 4, upper panel). With AP0 (i.e., the same approach proposed in Rüter [16] and applied by the majority of EU countries for the submissions for FMRL), the resulting sink in 2030 is equal to −22,942 Gg CO_2_ yr^−1^ i.e., 12% and 50% lower than the sink estimated by AP2 and AP1, respectively. As discussed above, the differences are mainly due to the increasing SW (for AP1) and PP domestic production (for AP1 and AP2) estimated by our analysis even with a constant harvest rate (Figure 3). The pattern highlighted by all the approaches (with an increasing sink from 2012 to 2020) is due to the temporary increase of DP estimated in this period (see for example the slope of the future DP estimated with AP1 in Figure 3), due to the difference between the historical DP in 2012 and the production (with AP1), DP (with AP2) or Inflow (with AP0) estimated in 2020. Between 2012 and 2020 we applied a linear regression between the last historical value and 2020 for all the approaches. After this date, all our approaches report a decreasing sink, with a quite similar trend (i.e., the slope of the lines in the upper panel of Figure 4, from 2020 to 2030).

The average historical HWP sink from 2000 to 2012 is equal to −44,731 Gg CO_2_ yr^−1^. This is about 10% of the sink by EU forest pools and nearly 1% of the total EU GHG emissions in the same period. In 2030, with a constant harvest scenario, the future HWP sink was reduced to −22,942 Gg CO_2_ yr^−1^ (with AP0), −34,901 Gg CO_2_ yr^−1^ (with AP2) and −25,652 Gg CO_2_ yr^−1^ (with AP2), i.e. -49% (with AP0), −22% (with AP1) and −43% (with AP2) compared with the historical average sink. This trend of decreasing HWP sink can be explained observing that, in the constant harvest scenario, the domestic production of each commodity (and the consequent inflow of C in the HWP pool) stabilizes and, as a consequence, the difference between the inflow and outflow tends to balance out. This means that with a constant harvest the HWP sink will eventually tend to zero, i.e. to “saturate”.

The lower panel in Figure 4 reports the historical and the future HWP sinks estimated, for each scenario, with AP0. In the increasing harvest scenario, the final HWP sink in 2030 (−43,172 Gg CO_2_ yr^−1^) is almost equal to the historical average HWP sink (2000–2012). This can be explained by the fact that the rate of increase of harvest assumed in this scenario is similar to the one observed in the previous period (see Figure 2). This means that in order to keep a constant HWP sink the rate of increase in future harvest (assuming a constant distribution of harvest to the various commodities) should not be lower than the rate of increase observed in the past.

As expected, reducing by 20% the future harvest rate, the 2030 sink decreases to −9,078 Gg CO_2_ yr^−1^ in 2030, i.e., −80% compared with the average historical sink. This is due to the cumulative effect of a reduced inflow to the HWP pool, to the annual decay rate affecting each commodity (i.e., the outflow) and to the quite strong reduction in the domestic production.

Despite our higher harvest scenario seems similar to the one followed in the EU Reference Scenario [20], results for HWP are not comparable due to different methodological assumptions. The main difference is that the EU Reference Scenario assumes that the HWP pool was in steady state in 2000 [33].

## Conclusions

The contribution of the forest sector to climate change mitigation results from different and partly competing options, such as increasing the forest sink or maximizing the energy or material substitution by wood products [6-9]. In this context, a better understanding of potential future carbon stock changes in the Harvest Wood Products pool is essential to define an effective mitigation strategy, capable to maximize the sum of the contribution of different mitigation options.

In this paper we estimated the CO_2_ emissions and removals in the HWP pool at EU level from 1990 to 2030, using the Tier 2 method from the 2013 IPCC KP Supplement and applying different scenarios of future harvest (and its distribution in different products).

The results of our study show that by assuming a constant historical harvest till 2030 the HWP sink at EU level will tend to decrease, for all the assumptions made on the distribution of harvest to different products. This is a consequence of the fact that, with a constant inflow of C in HWP pool, the HWP sink will sooner or later tend to saturate, i.e. to approach zero. A decreasing harvest will further speed up the tendency of HWP sink to approach zero. The current HWP sink will be maintained only by further increasing the current harvest in the future, as shown in our increasing harvest scenario. On the other hand, this latter scenario will tend to reduce the current sink in forest biomass, at least in the short term. Overall, our results suggest that there is limited potential for additional HWP sink in the EU.

Furthermore, our analysis suggests that other factors other than the total harvest may also play an important role in determining the future HWP emissions or removals. These factors include: (i) the relative share of different commodities such as furniture, plywood, paper and paper-like products, or energy; and (ii) the combined effect of production, import and export on the domestic production of each commodity. Depending on the specific country situation, in some case these factors may be even more important than the total amount of harvest in determining the future HWP emissions or removals. Therefore, when making projections on future HWP mitigation potential, the assumptions on (i) and (ii) above may play a crucial role.

Our results are based on possible correlations between harvest projections and subsequent HWP productions and do not consider technical and economic correlations between these variables. Looking to the material composition of each HWP commodity, a technical correlation between roundwood consumption (including production, import and export) used as feedstock for SW could be assessed.

Whereas in this paper we evaluated the HWP alone, from the analysis above it is clear that the HWP mitigation potential should be analyzed in conjunction with other mitigation components (e.g. sink in forest biomass, energy and material substitution by wood). To this aim, our future work will incorporate progressively the HWP into a broader modeling framework, including interactions with different options for forest management or the use of forest products for energy or material purposes (e.g., [6,25]).

## Methods

**Figure 5:**
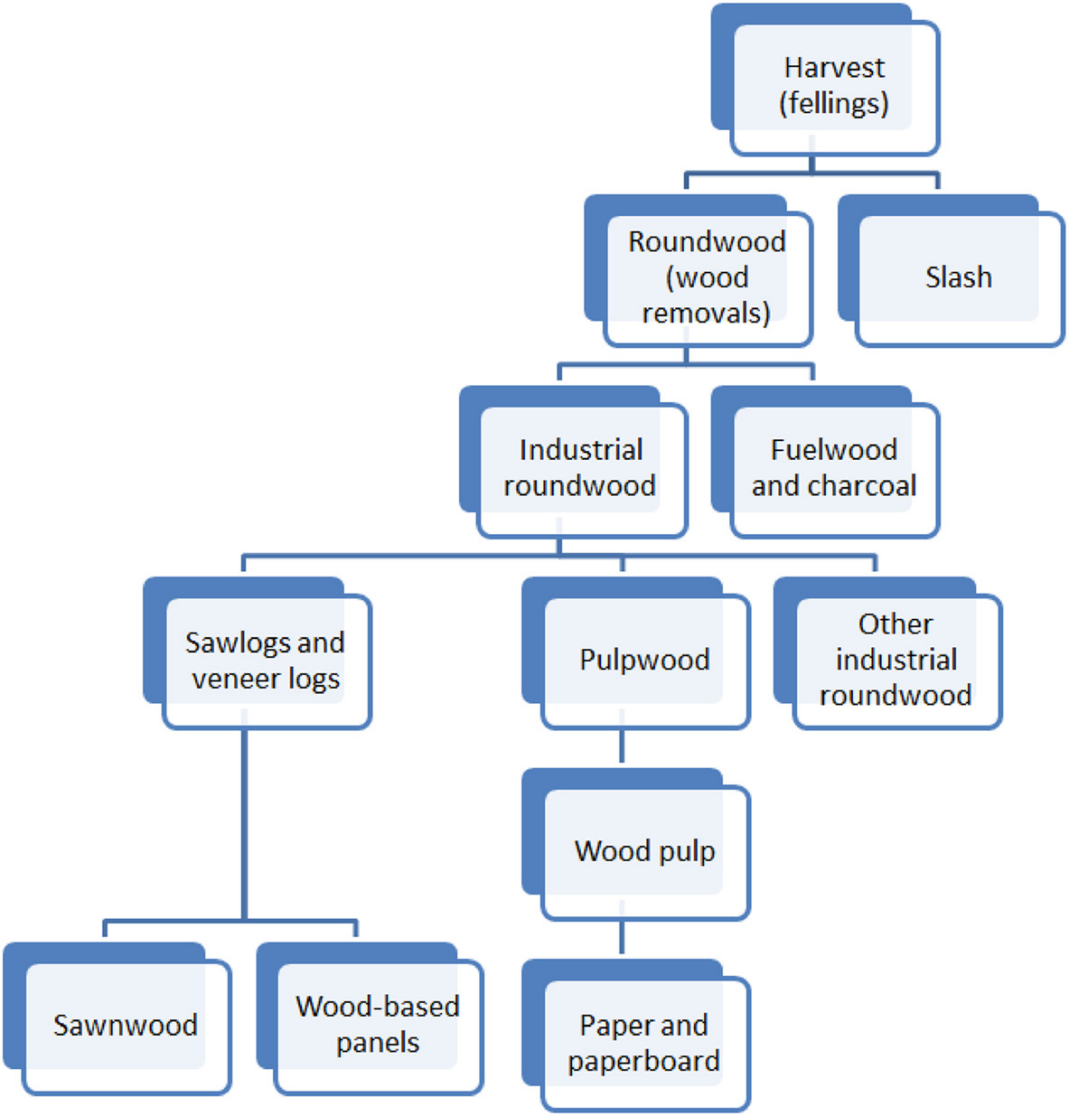
**Wood products FAO classification:** **classification of wood products based on FAO forest products definitions,** **adapted from 2013 IPCC KP Supplement** **[** 15 **].**

The IPCC Tier 2 method described by the 2013 Revised Supplementary Methods and Good Practice Guidance Arising from the Kyoto Protocol [15] involves the following steps:

1. Estimate the fraction of domestically produced commodities, distinguished between sawn wood, wood based panels and paper and paperboards (Figure 5). The share of domestic IRW originating from domestic forests in the overall consumption of IRW in year *i*, is computed considering the production (*IRW*_*P*_ and *PULP*_*P*_), imports (*IRW*_*IM*_ and *PULP*_*IM*_) and exports (*IRW*_*EX*_ and *PULP*_*EX*_) of industrial roundwood (IRW) and pulp (PULP), according to the following equations:

1 $$ {f}_{IRW}(i)=\frac{IR{W}_P(i)-IR{W}_{EX}(i)}{IR{W}_P(i)+IR{W}_{IM}(i)-IR{W}_{EX}(i)} $$

2 $$ {f}_{PULP}(i)=\frac{PUL{P}_P(i)-PUL{P}_{EX}(i)}{PUL{P}_P(i)+PUL{P}_{IM}(i)-PUL{P}_{EX}(i)} $$

Where, *f*_*IRW*_ is the share of industrial roundwood for the domestic production of HWP originating from domestic forests in year *i*; *f*_*PULP*_ is the share of domestically produced pulp for the domestic production of paper and paperboard in year *i*. The term in the denominator of Eq. (1) and (2 ^f^) equals the consumption.

2. Estimate the annual *Inflow* of C since 1900, applying the 2013 IPCC KP Supplement default conversion factors to the domestically produced commodities;
3. Estimate the annual *Outflow* of C, applying a first order decay function with constant annual default decay factors for each commodity;
4. Estimate the total C stock and C stock change.

This method can be applied not only to historic data series, but also to projections in the future.

Equations (1) and (2) were estimated using the original FAO units (i.e., m^3^ or Mg). Eq. (1) was applied to the IRW activity data (distinguished between conifers and non-conifers); Eq. (2) was applied to paper and paperboard.

The final annual HWP amount produced from domestic harvest is estimated as:



Where: *HWP*(*i*) is the harvested wood products amount produced from domestic harvest in year *i* for each commodity, in m^3^ (or Mg for paper) yr^−1^; *HWP*_*P*_(*i*) is the production of the particular HWP commodities (i.e., sawn wood, distinguished between conifers and non-conifers, wood based panels or paper and paper board) in year *i*, in m^3^ (or Mg for paper) yr^−1^. Eq. (3a) was applied to sawn wood and wood-based panels, Eq. (3b) to paper and paper board. In this last case we estimated an average share of domestic feedstock $\left(\overline{f_{IRW}(i)}\right)$ for the production of this specific commodity, equal to:

3 $$ \overline{f_{IRW}(i)}={f}_{IRW}{(i)}_{Con}\times {w}_{Con}+{f}_{IRW}{(i)}_{Non- Con}\times {w}_{Non- Con} $$

Where, *f*_*IRW*_(*i*)_*Con*_ and *f*_*IRW*_(*i*)_*Non*_-_*Con*_ are the shares of IRW estimated by Eq.(1) for conifers and non-conifers, respectively; *w*_*Con*_ and *w*_*Non*-*Con*_ are weighting factors derived by the total amount (i.e., production + import + export) of conifers and non-conifers, respectively.

### First-order decay functions

Inflow–outflow methods estimate the changes in carbon stocks by counting the amount of wood products into and out of the stock (FCCC/tP/2003/7). Changes in carbon stocks in year *i* are estimated on the basis of information (i) on the inflow of wood products into the stock and (ii) of assumed lifetimes and (iii) decay factors of these products (*Lifetime analysis*), according to the following equations (see Equations two-eight-five, 2013 IPCC KP Supplement and [34]):

4 $$ C\left(i+1\right)={e}^{-k}C(i) + \left[\frac{\left(1-{e}^{-k}\right)}{k}\right]* Inflow(i) $$

*C*(*i*) = carbon stock in the particular HWP category at the beginning of year *i*, in Gg C (default conversion factors are in Table 4).

**Table 4:** **Default conversion factors for HWP categories** (**based on** [15])

**HWP categories**	**Sawn wood**		**Wood based panels**	**Paper and paper boards**
	**Coniferous**	**Non**-**coniferous**		
**Conversion factor per air dry density**	0.225 Mg C m^−3^	0.280 Mg C m^−3^	0.269 Mg C m^−3^	0.386 Mg C Mg^−1^
**Default half**-**lives**	35 years		25 years	2 years

*k* = first-order decay constant for each HWP category, equal to ln(2)/*HL*, where *HL* is the half-life of each HWP pool in years (Table 4).

*Inflow*(*i*) = inflow to the particular HWP category during the year *i*, in Gg C yr^−1^.

The carbon stock change (*ΔC*(*i*) in Gg C yr^−1^) of the HWP category during the year *i*, is equal to:

5 $$ \varDelta C=C\left(i+1\right)-C(i) $$

Equations (4) and (5) were applied separately for each semi-finished wood products category (sawn wood coniferous and non-coniferous, wood-based panels and paper and paperboards). Finally (step 4, Figure 1), the total C stock and C stock change in HWP can be calculated for each country by summing up all the C stocks and C stock changes of all the commodities^d^.

### Approach 1 (AP1)

To apply the default Tier 2 IPCC method [15], the production of each commodity is needed. Moreover, to estimate the share of domestically produced industrial roundwood and pulp, import and export data are also requested. Therefore, in the first approach, we first looked for possible statistical correlations between the production, import and export quantities (i.e., the variables used in the first step described in Figure 1) and total roundwood (RW) and time (*t*, i.e., years) (Table 5). Based on a preliminary analysis of the results, the relations having a Pearson’s coefficient of correlation *r* > |0.69| were used to estimate the values of these parameters.

**Table 5:** **AP1 summary table**: **we estimated the statistical correlations** (**highlighted by the black crosses**) **between these categories** (**dependent variables**, ***y***) **and the following independent variables** (***x***: **total roundwood production** (**RW**) **and time** (**years**)) **using a simple linear model** ***y*** = ***a*** + ***b x***

**Correlations**	**Dependent variables**									**Independent variables**	
	**(** ***y*** **)**									**(** ***x*** **)**	
	**PRODUCTION**			**IMPORT**			**EXPORT**				
**KEY INPUT DATA**	**Total**	**C**	**NC**	**Total**	**C**	**NC**	**Total**	**C**	**NC**	**Total RW**	**Time**
**IRW and Pulp**		**X**	**X**		**X**	**X**		**X**	**X**	**X**	**X**
**Sawn wood**		**X**	**X**					**X**	**X**	**X**	**X**
**Wood based panels**	**X**									**X**	**X**
**Paper & paperboard**	**X**									**X**	**X**

Table 2 reports the correlations that were used for the linear regression to extrapolate production quantities into the future.

Depending on the highest resulting correlation, the future production of sawn wood (conifer and non-conifer), wood based panels and paper and paperboards and the import and export data requested by Eq. (1) and (2), were estimated with a simple linear model:

6 $$ y=a+bx $$

with *y* and *x* defined according to Table 5, and *a* and *b* defining the intercept and the slope of the function, respectively. The values of these parameters was estimated, for each country, using the *Proc Reg* procedure in the SAS® software, excluding possible outliers from the analysis of the distribution of the studentized residuals (i.e., the scaled version of residuals that are obtained by dividing each residual by its standard error) and evaluating the fitness of each model through the coefficient of determination *R* ^*2*^. The highest correlation coefficients were chosen for the regression, without taking into account the composition of feedstock of the particular HWP category. When no correlation could be established, an average of the previous 20 years was calculated and assumed to remain valid in future decades as well. For some countries, such as Belgium, Greece or Luxembourg (Table 1), where no data was available for the last 20 years, a shorter period (generally 10 years) was considered.

### Approach 2 (AP2)

With the second method, we check the statistical correlation between the domestic production of each commodity (i.e., the parameters used in the second step described above and reported in Figure 1), and both the industrial roundwood total production (*IRW*_*t*_) and time. Depending on the highest resulting correlation, the future domestic production of sawn wood (conifer and non-conifer), wood based panels and paper and paperboards can be estimated with the linear model reported in Eq. (6).

Coniferous and non-coniferous sawn wood are estimated as a function either of *IRW*_*t*_ or time, depending on the highest coefficient of correlation. If the correlation with one of these two categories is too low (i.e., based on a preliminary analysis of the data, r < |0.66|), but the total sawn wood (*SWt*) is above the threshold, then the difference between the *SWt* and the other commodity was used. If the correlation is valid only for *SWt*, we estimate the average amount of one commodity for the paste 1–2 decades (depending by the available country data), assume it remains valid for the future and then estimate the amount of the other commodity as the difference with *SWt*. If non correlation can be established, then the average amount of the sawn wood conifers (*SWc*) and sawn wood non-conifers (*SWnc*) for the past 10–20 years is applied as constant in the future as well.

The same approach is used for wood based panels (*WP*) and paper and paperboards (*PP*). Future productions are a function of either *IRWt* or time, depending on the highest correlation. If no correlation can be established, then the average of the past 10–20 years is assumed as valid for the future as well. Table 6 shows a synthesis of the method.

**Table 6:** **AP2 summary table**: ***SW***_***T***_ **was used as ancillary variable to estimate the** ***SW***_***NC***_ **or the** ***SW***_***C***_ **input data**

**Approach**	**(a)**	**(b)**		**(c)**		**(d)**
	**Eq. (6)**	**Eq. (6) + difference**		**Eq. (6) + Const. aver.**		**Constant average**
**Key data**	R^2^ > |0.66|	R^2^ > |0.66|	R^2^ > |0.66|	R^2^ > |0.66|	R^2^ > |0.66|	R^2^ > |0.66|
	for IRW_T_ or T	for IRW_T_ or T	for IRW_T_ or T	for IRW_T_ or T	for IRW_T_ or T	for IRW_T_ or T
**SW**_**C**_	**Yes**:	**Yes**:	**No**:	**No**:	**No**:	**No**:
	f(IRW_T_ or T)	f(IRW_T_ or T)	SW_T_-SW_NC_	Const. Average	SW_T_-SW_NC_	Const. Average
**SW**_**T**_	**Not used**	**Yes**:	**Yes**:	**Yes**:	**Yes**:	**Not used**
		f(IRW_T_ or T)	f(IRW_T_ or T)	f(IRW_T_ or T)	f(IRW_T_ or T)	
**SW**_**NC**_	**Yes**:	**No**:	**Yes**:	**No**:	**No**:	**No**:
	f(IRW_T_ or T)	SW_T_-SW_C_	f(IRW_T_ or T)	SW_T_-SW_C_	Const. Average	Const. Average
**WP**	**Yes**:					**No**:
	f(IRW_T_ or T)					Const. Average
**PP**	**Yes**:					**No**:
	f(IRW_T_ or T)					Const. Average

If the coefficient of determination (*R* ^*2*^) is > |0.66| (Yes in the table), a linear regression is used with either *IRW*_*T*_ or time. If no significant correlation can be established, the average of the past 20 years is used for the future (Constant average in the table).

## Endnotes

^a^This is the standing volume of all trees live or dead that are felled during a certain period, including those parts of trees that are not removed from the forest (harvest removals are a subset of fellings [35]).

^b^For forest products definitions, see: http://faostat.fao.org/Portals/_Faostat/documents/pdf/FAOSTAT-Forestry-def-e.pdf.

^c^i.e., “wood in the rough” which includes all wood in its natural state, used for wood products or for energy production (FAO, 2000).

^d^To convert carbon to CO_2_ multiply by (44/12).

^e^For many countries these data are the same.

^f^Please note that, according to GPG 2013 (Equation. two-eight-four) [15], *f*_*PULP*_ =0, if *f*_*PULP*_ <0. We assumed that *f*_*PULP*_ = 0 when (*PULP*_*P*_(*i*) − *PULP*_*EX*_(*i*)) = 0.

## Abbreviations

ARD: Afforestation, reforestation and deforestation
DP: Domestic production
AP2: Approach 2
FM: Forest management
HWP: Harvested wood product
IRW: Industrial roundwood
LULUCF: Land use, land use change and forestry
KP: Kyoto protocol
PCA: Principal component analysis
AP1: Approach 1
PP: Paper and paperboards
RL: Reference level
RW: Roundwood
SWc: Coniferous sawnwood
SWnc: Non-coniferous sawnwood
SWt: Total sawnwood
WP: Wood based panels
FRA: Forest resources assessment
NIR: National inventory report
